# 779. COVID-19 Pandemic and Catheter-associated Urinary Tract Infection Trends

**DOI:** 10.1093/ofid/ofab466.976

**Published:** 2021-12-04

**Authors:** Geehan Suleyman, Rita Kassab, Smitha Gudipati, Ramesh Mayur, Indira Brar

**Affiliations:** 1 Henry Ford Hospital, Detroit, Michigan; 2 Henry Ford Health System, Detroit, Michigan

## Abstract

**Background:**

It has been postulated that the COVID-19 pandemic would increase the overall catheter-associated urinary tract infections (CAUTI) risk in part due to higher acuity, increased indwelling urinary catheter (IUC) utilization, longer length of stay, changes in infection prevention practices due to staffing shortages. However, reported data are limited. The goal of this study was to evaluate the impact of the COVID-19 pandemic on our CAUTI rates.

**Methods:**

This was a retrospective cross-sectional study comparing CAUTI rate per 1,000 indwelling urinary catheter (IUC) days, urine culture (UC) utilization rate per 1,000 IUC days, IUC utilization rate per 1,000 patient days, Standardized Infection Ratio (SIR) and Standardized Utilization Ratio (SUR) in the pre-COVID-19 period from January 1, 2019 to December 31, 2019 to the COVID-19 period from April 1, 2020 to March 31, 2021 at an 877-bed tertiary care hospital in Detroit, Michigan. CAUTI, UC utilization and IUC utilization rate were extracted from the electronic medical record (Epic™ Bugsy). SIR and SUR data were extracted from National Healthcare Safety Network (NHSN).

**Results:**

The average CAUTI rate per 1,000 IUC days decreased from 0.99 pre-COVID-19 to 0.64 during COVID-19, yielding a 35% reduction. The UC order rate per 1,000 IUC days decreased from 19.19 to 18.83 with only 2% reduction. However, IUC utilization rate increased by 55% from 0.184 to 0.286. The SIR decreased from 0.483 to 0.337 with a 30% reduction, although this was not statistically significant ((P-value 0.283). The overall SUR decreased significantly from 0.806 to 0.762 (P-value < 0.001). Figure 2 is a control chart of the CAUTI rate from July 2019 to April 2021.

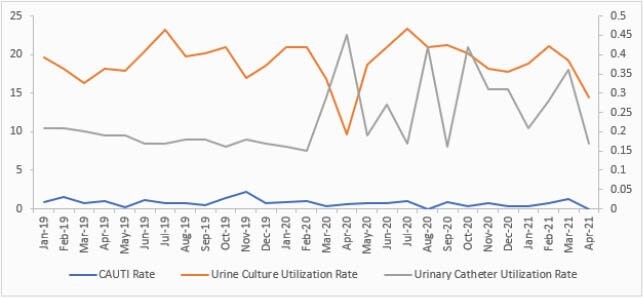

Figure 1. CAUTI, indwelling urinary catheter and urine culture utilization rates pre-and during COVID-19 pandemic.

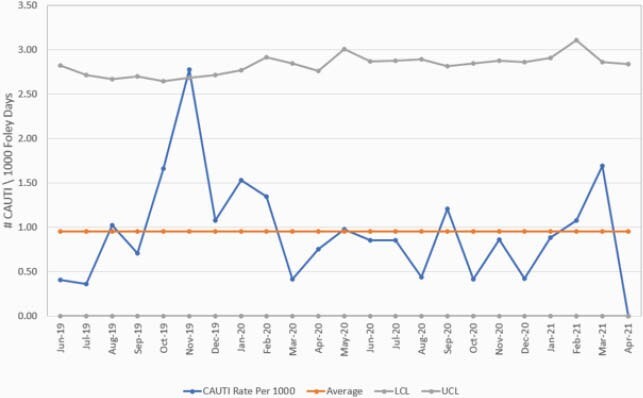

Figure 2. CAUTI control chart pre-and during COVID-19 pandemic.

**Conclusion:**

Although the IUC utilization increased during the COVID-19 pandemic, CAUTI rate, SIR and SUR decreased and UC orders remained unchanged. Thus, the pandemic did not have a negative impact on our CAUTI rates.

**Disclosures:**

**All Authors**: No reported disclosures

